# Students’ Manual of Diseases of the Nose and Throat

**Published:** 1883-12

**Authors:** 


**Students' Manual of Diseases of the Nose and Throat.** A digest, descriptive of the more commonly seen diseases of the upper air tract, with the methods of their treatment. By J. M. W. KITCHEN, M. D., Assistant Surgeon Metropolitan Throat Hospital. New York: G. P. Putnam's Sons. 1883.

A small book of 124 pages, giving, however, an excellent resume of the subject. It contains what should be known by every graduate in medicine, but omits the rarer diseases of the upper air tract. The publishers have issued the book in a handsome form.

